# Antibody-mediated phagocytosis contributes to the anti-tumor activity of the therapeutic antibody daratumumab in lymphoma and multiple myeloma

**DOI:** 10.1080/19420862.2015.1007813

**Published:** 2015-03-11

**Authors:** Marije B Overdijk, Sandra Verploegen, Marijn Bögels, Marjolein van Egmond, Jeroen J Lammerts van Bueren, Tuna Mutis, Richard WJ Groen, Esther Breij, Anton CM Martens, Wim K Bleeker, Paul WHI Parren

**Affiliations:** 1 Genmab; Utrecht, The Netherlands; 2 Department of Molecular Cell Biology and Immunology, VU University Medical Center, Amsterdam, The Netherlands; 3 Department of Surgery; VU University Medical Center; Amsterdam, The Netherlands; 4 Department of Clinical Chemistry and Hematology; University Medical Center; Utrecht, The Netherlands; 5 Department of Cell Biology; University Medical Center; Utrecht, The Netherlands; 6 Department of Immunology; University Medical Center; Utrecht, The Netherlands; 7 Department of Cancer and Inflammation Research, Institute of Molecular Medicine, University of Southern Denmark, Odense, Denmark; 8 Department of Immunohematology and Blood Transfusion; Leiden University Medical Center; Leiden, The Netherlands

**Keywords:** macrophage, phagocytosis, therapeutic antibody, CD38, daratumumab, multiple myeloma, Burkitt's lymphoma

## Abstract

Daratumumab (DARA) is a human CD38-specific IgG1 antibody that is in clinical development for the treatment of multiple myeloma (MM). The potential for IgG1 antibodies to induce macrophage-mediated phagocytosis, in combination with the known presence of macrophages in the tumor microenvironment in MM and other hematological tumors, led us to investigate the contribution of antibody-dependent, macrophage-mediated phagocytosis to DARA's mechanism of action. Live cell imaging revealed that DARA efficiently induced macrophage-mediated phagocytosis, in which individual macrophages rapidly and sequentially engulfed multiple tumor cells. DARA-dependent phagocytosis by mouse and human macrophages was also observed in an in vitro flow cytometry assay, using a range of MM and Burkitt's lymphoma cell lines. Phagocytosis contributed to DARA's anti-tumor activity in vivo, in both a subcutaneous and an intravenous leukemic xenograft mouse model. Finally, DARA was shown to induce macrophage-mediated phagocytosis of MM cells isolated from 11 of 12 MM patients that showed variable levels of CD38 expression. In summary, we demonstrate that phagocytosis is a fast, potent and clinically relevant mechanism of action that may contribute to the therapeutic activity of DARA in multiple myeloma and potentially other hematological tumors.

## Abbreviations

ADCC: antibody-dependent cellular cytotoxicity
BM: bone marrow
BL: Burkitt's lymphoma
CDC: complement-dependent cytotoxicity
CCS: cosmic calf serum
DARA: daratumumab
DP: double positive
E:T: effector to target ratio
FcγR: Fc-gamma receptor
IMiD: immunomodulatory drug
Mφ: macrophage
mAb: monoclonal antibody
MNC: mononuclear cells
MM: multiple myeloma
PBMC: peripheral blood mononuclear cells

## Introduction

Phagocytosis is an efficient and fast mechanism for the elimination of pathogens and apoptotic cells. Phagocytosis can be induced through several pathways, including recognition of surface-bound antibodies (Ab), complement factors or pathogen-associated molecular patterns. Antibody-dependent phagocytosis of IgG1-opsonized pathogens as well as cancer cells occurs via binding to Fcγ-receptors (FcγRs), specifically via the low-affinity receptors FcγRIIa and FcγRIIIa.^1,2^ Macrophages (mφ), representing professional phagocytes, are abundant in tumor stroma^3-5^ and phagocytosis by mφ might therefore be a very potent mechanism of action of therapeutic Ab in cancer treatment.

By using mouse strains deficient in specific leukocyte subpopulations or by depleting specific effector cell subsets, mφ were shown to represent the main effector cells in the anti-tumor activity of CD20-targeting monoclonal Ab (mAb) in vivo.^6,7^ Furthermore, for SGN-30 (chimeric IgG1 CD30 mAb), SGN-40 (humanized IgG1 CD40 mAb) and a humanized CD70 mAb, all of which were shown to mediate phagocytosis in vitro, mφ were shown to be the major effector cells in vivo.^8-10^

Daratumumab (DARA) is a human IgG1 mAb targeting CD38, a 46-kDa type II transmembrane glycoprotein that is expressed at high levels on malignant cells in multiple myeloma (MM).^11^ DARA was granted Breakthrough Therapy Designation by the Food and Drug Administration for MM patients who have received at least 3 prior lines of therapy including a proteasome inhibitor and an immunomodulatory agent, or patients double refractory to these agents, in 2013, and it is currently in multiple Phase 3 clinical trials for the treatment of MM. DARA can induce tumor cell killing through a number of effector mechanisms, including the Fc-dependent effector mechanisms complement-dependent cytotoxicity (CDC) and natural killer (NK)-cell mediated antibody-dependent cellular cytotoxicity (ADCC).^12^ Macrophages are known to be abundantly present in the bone marrow of MM patients,^4,5^ and macrophage-mediated phagocytosis has been demonstrated to be induced by several mAbs targeting MM cells.^13-15^ The capacity of DARA to induce macrophage-mediated phagocytosis has not been studied thus far.

Here, we explored the capacity of DARA to kill tumor cells through antibody-dependent phagocytosis. DARA-dependent phagocytosis of Burkitt's lymphoma (BL) and MM cell lines in vitro was explored using live cell imaging and flow cytometry. Furthermore, DARA-dependent phagocytosis of patient-derived MM cells was studied ex vivo. Finally, the contribution of phagocytosis to the anti-tumor activity of DARA in vivo was studied using an isotype variant of DARA that does not induce phagocytosis in the presence of mouse macrophages. Our results showed that phagocytosis contributes to the anti-tumor activity of DARA in vitro and in vivo.

## Results

### DARA induces phagocytosis of CD38-positive tumor cells

**Figure 1.**
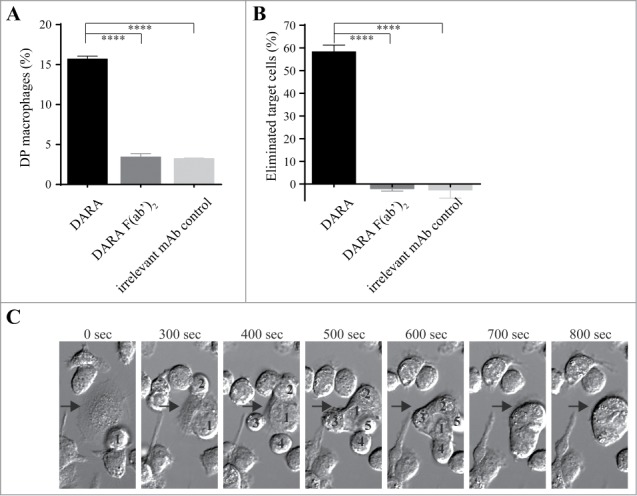
*Flow cytometry and live cell imaging reveals macrophage-mediated phagocytosis of CD38+ tumor cells in the presence of DARA.* Co-cultures of mouse mφ and Daudi cells in the presence of 6.7 nM DARA or F(ab’)_2_ fragments thereof, E:T ratio of 1:1 (**A, B**) or 3:1 (**C**). (**A**) Double positive (DP) mφ were characterized as F4/80^+^calcein^+^CD19^–^ and the percentage DP macrophages was calculated as described in Materials and Methods. (**B**) The percentage eliminated target cells was calculated from the number of remaining F4/80^-^ cells as described in Materials & Methods. Each bar shows mean ± SEM, results from a representative experiment are shown (*n* = 3). (**C**) Time-lapse imaging microscopy, bright field images of a mouse mφ (arrow) that sequentially engulfed 5 individual Daudi cells (numbers) over a period of 800 s. The images are representative for observations in multiple independent phagocytosis experiments (*n* = 3) (****p < 0.0001 Bonferroni's multiple comparison test).

To explore the induction of phagocytosis by DARA, we set up a flow cytometric phagocytosis assay using mouse mφ as effector cells and Burkitt's lymphoma (BL) Daudi cells as target cells. Phagocytosis was assessed in 2 ways: 1) by determining the percentage of double positive (DP) mφ (representative flow cytometry plots shown in **Fig. S1)** and 2) by determining the percentage of eliminated target cells (calculated as described in Materials & Methods). DARA induced macrophage-mediated phagocytosis, as shown by an increase in the number of DP mφ (**Fig. 1A**) and elimination of a substantial proportion of target cells (**Fig. 1B**). Live cell imaging confirmed that this increase in DP mφ and eliminated target cells was indeed due to DARA-dependent phagocytosis. Supplemental movie 1 shows time-lapse imaging microscopy of co-cultures of DiO (green) labeled mouse mφ and DiB (blue) labeled Daudi cells in the presence of DARA. All target cells visible in the field of observation had been phagocytosed at the end of the experiment. Interestingly, using time-lapse imaging microscopy, we frequently observed mφ to engulf multiple DARA-opsonized target cells in a relatively short time span. **Fig. 1C** and Supplemental movie 2 show an individual mouse mφ (designated with an arrow) that sequentially engulfed 5 Daudi cells within a 15 min. period. In the presence of an irrelevant antibody control, no engulfment was observed (Supplemental movie 3). These real-time data show that phagocytosis induction is a very rapid and efficient mechanism of action of DARA.

### Threshold CD38 expression level for phagocytosis induction

To explore the effect of CD38 expression levels on phagocytosis induction by DARA, we set up a flow cytometric assay with mouse mφ and leukemic target cells with variable levels of CD38 expression (**Table 1**). In a pilot experiment, we found that the MM cell lines UM9 and L363, with relatively low CD38 expression (50,000∼100,000 and 100,000∼150,000 molecules/cell, respectively) were not susceptible to DARA-dependent phagocytosis. However, uptake into mφ and substantial elimination of target cells was consistently observed for CD38-transduced UM9-CD38 and L363-CD38 variants with high levels of CD38 expression (350,000∼600,000 and 450,000∼800,000 molecules/cell, respectively). These results suggest that DARA-dependent phagocytosis is related to CD38 expression levels. However, it is difficult to define a threshold level of CD38 expression that allows efficient DARA-dependent phagocytosis, as phagocytosis was also consistently observed in Wien-133 cells that express relatively low CD38 levels (**Table 1**). In addition, large differences, especially in the percentage of eliminated target cells, were observed between cell lines with comparable CD38 expression levels (e.g., Daudi and Raji, **Table 1**). Thus, additional factors are likely to determine the efficacy of DARA-dependent phagocytosis.

**Table 1.** DARA-dependent mφ-mediated phagocytosis of human multiple myeloma and lymphoma cell lines

Cell Line	CD38 range (molecules/cell)	DP mφ range(%)	Elimination range(%)
Wien-133	100,000 ∼ 150,000	5–20	0–40
Raji	150,000 ∼ 350,000	7–15	0–10
Ramos	200,000 ∼ 300,000	12–25	0–20
Daudi	200,000 ∼ 400,000	12–40	29–79
UM9-CD38	350,000 ∼ 600,000	5–8	2–50
L363-CD38	450,000 ∼ 800,000	9–10	4–70

Ranges based on at least 3 independent experiments.

### Phagocytosis contributes to the anti-tumor activity of DARA in vivo

**Figure 2.**
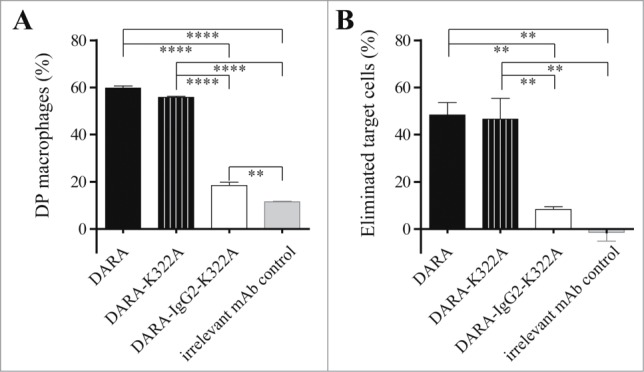
*Induction of macrophage-mediated phagocytosis is strongly impaired for an IgG2 isotype variant of DARA.* Phagocytosis of Daudi cells by mouse mφ in the presence of 6.7 nM mAb, E:T ratio of 1:1. (**A**) Double-positive (DP) mφ were characterized as F4/80^+^calcein^+^CD19^–^ and the percentage DP macrophages was calculated as described in Materials and Methods. (**B**) Percentage eliminated target cells was calculated using the number of remaining F4/80^-^ cells as described in Materials & Methods. Each bar shows mean ± SEM, results from a representative experiment (*n* = 3) (**p < 0.01, ****p < 0.0001 Bonferroni's multiple comparison test).

We previously demonstrated that, in contrast to human IgG1, the human IgG2 isotype shows weak to no phagocytosis activity with mouse mφ.^16^ Therefore, we compared DARA to a DARA-IgG2 isotype variant to study the contribution of phagocytosis to the in vivo efficacy of DARA in mouse xenograft tumor models. To restrict in vivo effector cell activity to mouse mφ, we made use of immune-deficient SCID-BEIGE mice, which lack B, T and NK cells. ADCC mediated by mφ is not expected to contribute in vivo, as we did not observe extracellular lysis after 24 h incubation of Daudi cells with mφ in the presence of DARA (**Fig. S3**). To exclude CDC, a known effector mechanism of DARA, Fc mutants were generated in which the lysine residue at position 322 was mutated to alanine (referred to as DARA-K322A and DARA-IgG2-K322A). Duncan et al. and Idusogie et al. showed K322 to be a critical residue for human C1q binding and complement activation,^17,18^ and we recently confirmed that the K322A mutation also leads to strongly reduced binding of mouse C1q.^19^ The K322A mutation itself did not affect the capacity of DARA to induce macrophage-mediated phagocytosis in vitro, as shown by similar percentages of both DP mφ and eliminated target cells induced by DARA and DARA-K322A (**Fig. 2A and B**). The percentage of DP mφ was strongly reduced when the DARA-IgG2-K322A variant was used instead of DARA or DARA-K322A. In addition, the percentage of eliminated target cells was significantly lower with the DARA-IgG2-K322A variant. This confirms that phagocytic capacity was preserved in DARA-K322A, whereas DARA-IgG2-K322A showed strongly impaired phagocytic capacity.

**Figure 3.**
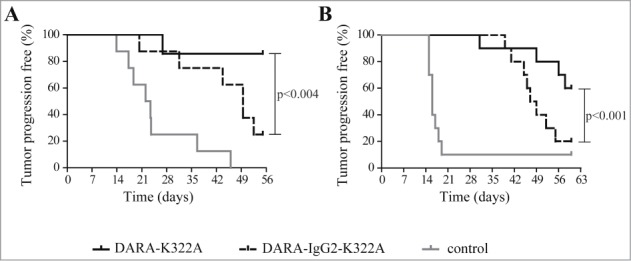
*Phagocytosis contributes to the in vivo anti-tumor effect of DARA.* (**A**) Kaplan-Meier plot showing time to tumor progression (cutoff set at a tumor volume > 800 mm^3^) for mice that had been inoculated s.c. with 20 × 10^6^ Daudi-luc cells (8 mice per group). Subsequently, mice were treated i.p. with 250 μg mAb per mouse (∼12.5 mg/kg) at day 0. Tumor progression was significantly reduced in DARA-K322A-treated mice compared to DARA-IgG2-K322A treatment (*p* < 0.004 Mantle-Cox log-rank test at time to progression). (**B**) Kaplan-Meier plot showing time to tumor progression (cutoff set at bioluminescence > 50 000 cpm) for mice that had been inoculated i.v. with 2.5×10^6^ Daudi-luc cells (10 mice per group). Subsequently, mice were treated i.p. with 10 μg mAb per mouse (∼0.5 mg/kg) at day 0. Tumor progression was significantly reduced in DARA-K322A-treated vs. DARA-IgG2-K322A-treated mice (*p* < 0.001 Mantle-Cox log-rank test at time to progression).

In a subcutaneous Daudi-luc tumor xenograft model, DARA-K322A provided significantly stronger inhibition of tumor growth than DARA-IgG2-K322A (**Fig. 3A**), indicating an important contribution of phagocytosis to the in vivo efficacy of DARA. Furthermore, in the intravenous leukemic Daudi-luc xenograft model, in which mice were treated at the time of tumor challenge, DARA-K322A also demonstrated a significantly stronger tumor growth inhibition compared to DARA-IgG2-K322A (**Fig. 3B**). Upon therapeutic treatment in this leukemic Daudi-luc xenograft model, DARA-K322A also showed better potency than DARA-IgG2-K322A (treatment with 0.5 mg/kg at day 14), as shown in **Fig. S2**. These data demonstrate that phagocytosis contributes to the in vivo mechanism of action of DARA.

### Patient MM tumor cells are efficiently phagocytosed by human macrophages in presence of DARA

**Figure 4.**
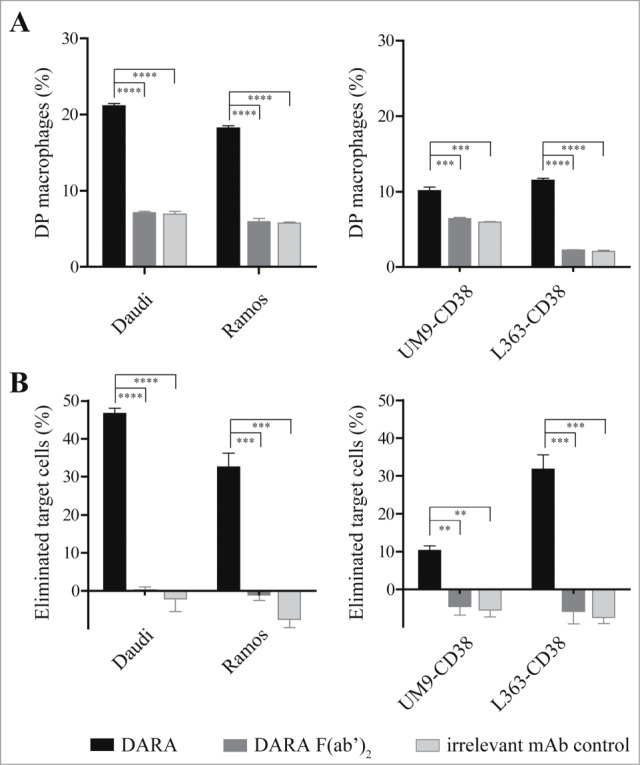
*Human macrophages induce DARA-dependent phagocytosis of BL and MM cell lines.* Phagocytosis of BL cells (left panel) or MM cells (right panel) by human mφ in the presence of 6.7 nM mAb, E:T ratio of 2:1. (**A**) Percentage of double positive (DP) mφ characterized as CD11b^+^calcein^+^target Ag^–^. (**B**) Percentage of eliminated target cells calculated from the number of remaining CD11b^-^ cells as described in Materials & Methods. Each bar shows mean ± SEM, results from a representative experiment (*n* = 3) (**p < 0.01, ***p < 0.001, ****p < 0.0001 Bonferroni's multiple comparison test).

**Figure 5.**
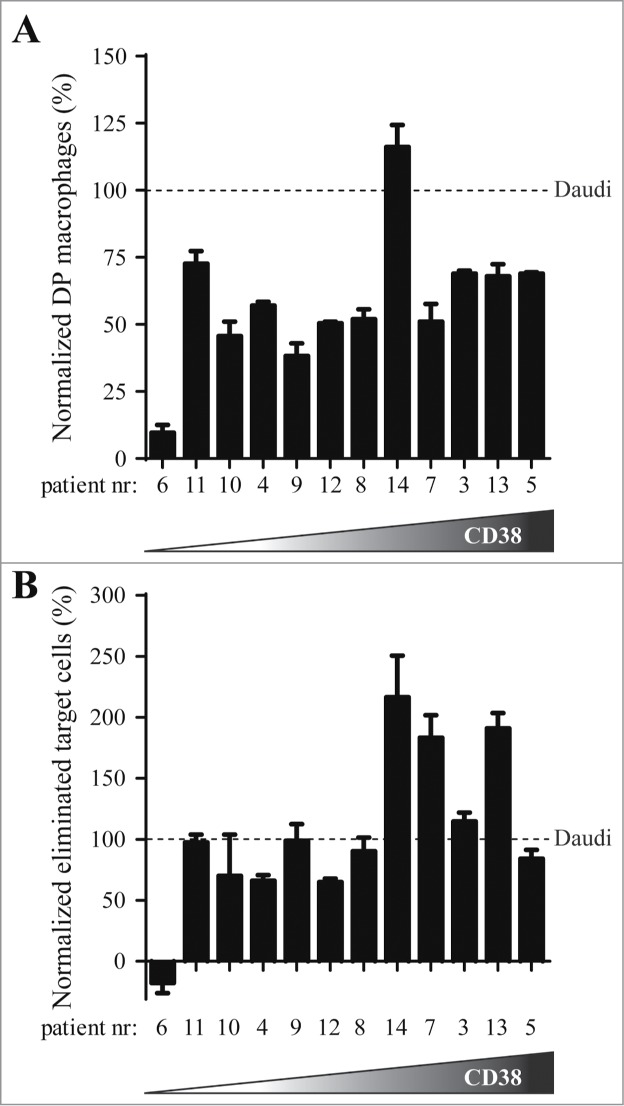
*DARA-dependent phagocytosis of patient MM cells by human macrophages.* Patient MM cells were incubated with human mφ, obtained from healthy donors (E:T ratio 2:1), in the presence of 6.7 nM mAb. To correct for differences in potency between batches of human donor mφ obtained from different donors, results were normalized by calculating the ratio of patient MM cells to an internal Daudi cell standard (observed in the same experiment). The 12 patient samples are ranked according to their CD38 expression level, with cells from patient 6 exhibiting lowest (10,000 molecules per cells) and patient 5 highest (550,000 molecules/cell) CD38 expression. (**A**) Normalized percentage of DARA-specific double positive (DP) mφ. **(B)** Normalized percentage of DARA-specific eliminated target cells. Each bar shows mean ± SEM of triplicates.

To translate our observations from xenograft tumor models to patients, we explored DARA-dependent phagocytosis of patient MM cells with human mφ. Monocytes isolated from healthy donors were cultured in the presence of granulocyte-macrophage colony-stimulating factor (GM-CSF) and characterized as CD11b^+^CD64^+^CD32^+^CD16^+/-^ mφ (data not shown). First, we explored the efficacy of phagocytosis by human mφ in presence of DARA using BL cell lines Daudi and Ramos, or MM cell lines UM9-CD38 and L363-CD38. Again, phagocytosis was assessed by determining the percentage DP mφ and percentage eliminated target cells. **Fig. 4A and B** show the percentage DP mφ and eliminated target cells, respectively, using BL and MM cell lines. Human mφ enhanced phagocytosis of DARA-opsonized tumor cells relative to background levels in the presence of DARA F(ab’)_2_ fragments or irrelevant mAb control. Second, we explored DARA-dependent phagocytosis of patient MM cells obtained from either bone marrow (BM), pleural fluid or blood. The MM patient samples contained more than 60% plasma cells, characterized as CD138-positive cells. CD38 expression ranged from 10,000 to 550,000 molecules/cell (**Table 2**). Peripheral blood, from 7 different healthy donors for which the FcγRIIa and IIIa-polymorphism were determined (**Table 2**), was used as a source of human mφ. As the potency of the human mφ differed between experiments, most likely due to differences in macrophage batches obtained from different donors, Daudi cells were included in each experiment as an internal control (see Materials & Methods). **Fig. 5A and B** show normalized percentages of DP mφ and eliminated target cells, respectively, calculated from the ratio of patient MM cells and the internal Daudi cell standard. For 11 of 12 patients, efficient DARA-dependent phagocytosis was observed, except for patient 6, for which MM cells expressed very low levels of CD38 (∼10,000 molecules/cell) that were found insensitive to DARA-dependent phagocytosis. Based on these observations we conclude that phagocytosis is a relevant and powerful effector mechanism of DARA, and for patient-derived MM cells.

**Table 2.** Characteristics of MM patient samples and mφ donors used in phagocytosis experiments

Characteristics MM patient samples			Characteristics mφ donors used in phagocytosis experiments	
Patient nr	Origin of MM cells	CD38 (molecules/cell)	FcγRIIa polymorphism*	FcγRIIIa polymorphism*
6	Blood	∼10,000	131H/R	158V/F
11	BM	∼35,000	131H/R	158V/F
10	BM	∼60,000	131H/R	158F/F
4	BM	∼70,000	131H/R	158V/F
9	BM	∼80,000	131H/R	158F/F
12	BM	∼95,000	131H/H	158V/F
8	BM	∼100,000	131H/R	158F/F
14	BM	∼105,000	131H/R	158V/F
7	BM	∼220,000	131H/R	158V/F
3	BM	∼230,000	131H/H	158V/F
13	BM	∼255,000	131H/R	158F/F
5	Pleural fluid	∼550,000	131H/R	158V/F

* 131H/R, heterozygous; 131H/H, homozygous; 158V/F heterozygous; 158F/F, homozygous BM = bone marrow.

## Discussion

In this study we investigated the capacity of DARA to induce macrophage-mediated phagocytosis and we observed that mouse and human mφ phagocytosed several CD38-positive BL and MM cell lines in the presence of DARA. Importantly, DARA was able to induce human macrophage-mediated phagocytosis of MM cells from 11 of 12 MM patients. Phagocytosis of patient MM cells by human mφ appeared to be more efficient than for the MM cell lines. Finally, phagocytosis was shown to contribute to the anti-tumor activity of DARA in vivo, in 2 different xenograft models.

Time-lapse imaging microscopy studies demonstrated that some macrophages engulfed multiple DARA-opsonized target cells, whereas other macrophages did not show phagocytic capacity. Comparison of different effector-to-target ratios revealed that, in all cases, only a maximum of ∼40% of mouse mφ actively engulfed one or multiple target cells (data not shown). Examining different target cell lines, we observed that the percentage of DP mφ was more or less comparable for all cell lines, whereas there was a striking difference in the percentage of eliminated target cells. This difference might be due to the number of target cells engulfed per mφ or it might be due to a difference in rate of phagocytosis between cell lines. Overall, the combination of the percentage of DP macrophages and the percentage of eliminated target cells provided an adequate representation of phagocytic capacity.

Although the capacity of DARA to induce phagocytosis was to some extent related to CD38 expression levels on target cells, differences in phagocytosis-mediated elimination of different target cell lines could not solely be explained by the differences in CD38 expression level, which confirms previous findings of Leidi et al.^20^ They compared phagocytosis of rituximab (chimeric IgG1 CD20 mAb) opsonized B-chronic lymphocytic leukemia and mantle cell lymphoma cells by human mφ and found that, although CD20 expression was required for rituximab-dependent phagocytosis, CD20 expression levels did not correlate with phagocytosis activity, as was also observed for ADCC.^20,21^ In contrast, target expression was previously shown to significantly affect CDC.^21,22^ Target cell size and shape have been suggested to influence phagocytosis efficacy and a model was proposed for size-dependent transitions that may determine whether a target cell will be phagocytosed or not.^23,24^ Cell size of the different BL and MM cells used in our study appeared to be comparable; however, difference in shape might account for the difference in phagocytosis efficiency. In addition to target expression levels, target cell size and shape, so-called ‘don't eat me’ signals on target cells play an important role in regulating phagocytosis. A prominent example is CD47, which inhibits phagocytosis via ligation to its receptor SIRPα on the phagocytic cell.^25^ CD47 is described to be upregulated on leukemic cells to avoid phagocytosis,^26^ which can be counterbalanced by the expression of calreticulin that gives a pro-phagocytic signal upon receptor binding.^27,28^ Human CD47 is not able to bind to mouse SIRPα,^29,30^ and therefore CD47 cannot account for the difference in phagocytosis efficiency with mouse mφ on the different target cell lines. However, CD47 expression may have influenced phagocytosis efficiency with human mφ. Overall, there might be other, yet undefined, regulators of phagocytosis and this possibility needs further investigation.

In vivo studies that address the role of mφ contributing to the mechanism of action of mAbs via mφ depletion, cannot discriminate between macrophage-mediated phagocytosis and macrophage-mediated ADCC. Therefore, we compared the efficacy of DARA with a matched IgG2 isotype variant that did not induce phagocytosis by mouse mφ. DARA induced significantly stronger tumor growth inhibition compared to the IgG2 variant in both a subcutaneous as well as a leukemic intravenous Daudi-luc xenograft model. Upon therapeutic treatment in the leukemic Daudi-luc xenograft model, DARA was also more potent compared to the IgG2 variant. Because macrophage-mediated ADCC is not expected to contribute to the anti-tumor effect of DARA and DARA-IgG2 (**Fig. S3** and Reference 16), these experiments confirm a significant role for macrophage-mediated phagocytosis in vivo. It is noted that DARA-IgG2 still induced significantly stronger tumor growth inhibition compared to the control in both models. As (1) a contribution of CDC was excluded in our experiments by using complement activation-deficient K322A mutant, and (2) a contribution of ADCC mediated by NK cells was excluded by selecting SCID-BEIGE lacking B, T and NK cells, potential other anti-tumor effects of DARA and DARA-IgG2 require further study. Such mechanisms could include the modulation of CD38 ectoenzyme function^31^ by DARA or the induction of polymorphonuclear neutrophil-mediated ADCC, which was shown to be similar for IgG1 and IgG2 antibodies targeting epidermal growth factor receptor.^16,32^

Overall, our results demonstrated a role for mφ-mediated phagocytosis in the in vivo mechanism of action of DARA in preclinical models. The contribution of phagocytosis to the in vivo efficacy for CD20-targeting mAb was recently shown. CD20 mAb treatment resulted in depletion of B cells in blood and liver within 2 hours.^33^ After partial hepatectomy, the efficiency of B-cell depletion in the blood was reduced, indicating that the liver contributes to the B-cell depletion in blood. Intravital imaging demonstrated depletion of B cells in the liver via phagocytosis by resident liver mφ (Kupffer cells). With live cell imaging in vitro*,* we also observed that DARA-dependent phagocytosis is a very fast mechanism. Delineation of the site of DARA-mediated phagocytosis in vivo, either in situ by tumor macrophages or via the reticuloendothelial system, requires further investigation.

In view of the high potential of DARA in MM, we explored the efficacy of DARA-dependent macrophage-mediated phagocytosis on patient MM cells. Potent DARA-dependent phagocytosis of MM cells was observed in 11 of 12 patient samples ex vivo, indicating that phagocytosis is a potential mechanism of action for DARA. The normalized results for DP mφ was lower than 100 percent for most of these MM patients, indicating that the percentage of macrophages that showed phagocytic activity was lower with MM patient cells than with Daudi cells. In contrast, the normalized percentage of eliminated target cells was 100 percent or higher in half of the patients, suggesting that the patient MM cells are more efficiently or rapidly phagocytosed than the Daudi cells. MM cells from one patient with low CD38 expression (∼10,000 molecules/cell) were not susceptible to DARA-dependent phagocytosis. The FcγRIIa 131 and FcγRIIIa 158 polymorphisms of donors used were not found to affect efficacy of phagocytosis by DARA. The observed susceptibility of MM cells might raise the question whether normal healthy white blood cells with low CD38 expression might also be susceptible to DARA-mediated phagocytosis. However, we previously showed that monocytes and NK-cells from peripheral blood mononuclear cells of a healthy donor were not killed by DARA.^34^

For MM patient therapy, the current focus is on combination therapy of mAb with immunomodulatory drugs (IMiDs).^37,38^ IMiDs currently used, e.g., lenalidomide and pomalidomide, are described to enhance NK-cell mediated ADCC.^39^ Combining DARA with lenalidomide enhanced ADCC-mediated elimination of MM cells.^34^ Macrophages in the BM microenvironment of MM cells, so-called tumor-associated mφ, have been shown to play an important role in supporting MM cell growth and survival.^4^ Furthermore, these mφ may protect MM cells from chemotherapy drug-induced apoptosis.^5^ Remarkably, tumor-associated mφ may still induce Fc-dependent anti-tumor activity,^40^ supporting a role for DARA-induced phagocytosis in the BM. Another approach is the combination with drugs regulating anti-phagocytic signals. Anti-CD47 mAb has been shown to synergize with rituximab in the treatment of non-Hodgkin lymphoma.^41^ Moreover, targeting the CD47-SIRP α axis has now been shown to increase efficacy of several therapeutic antibodies.^42,43^ CD47 is described to be highly expressed on CD38^+^ MM cells,^44^ suggesting that inhibition of the CD47-SIRP α axis might be an interesting therapeutic approach in combination with DARA.

Overall, we have shown that DARA induces macrophage-mediated phagocytosis of BL and MM cancer cell lines and MM patient cells. The enhanced efficacy of DARA-K322A versus DARA-IgG2-K322A in a subcutaneous and a leukemic intravenous xenograft tumor model suggests that phagocytosis also contributes to the efficacy of DARA in vivo. We conclude that phagocytosis is a rapid and potent mechanism of action that may contribute to the therapeutic activity of DARA in MM patients.

## Materials and Methods

### Cell lines

Raji, Ramos and Daudi-cells (human BL) were obtained from the ATCC (CCL-86, CRL-1596 and CCL-213, respectively). Wien-133 cells were kindly provided by Dr. Geoff Hale (BioAnaLab Limited, Oxford, UK). Daudi cells were transfected with gWIZ luciferase as previously described^16^ (Daudi-luc). The MM cell lines UM-9, generated at the University Medical Center (Utrecht, the Netherlands),^45^ and L363, obtained from the ATCC and gene-marked with GFP and luciferase marker genes,^46^ were transduced with human CD38 gene to obtain CD38 expression levels comparable to primary myeloma cells. For this, the amphotropic Phoenix packaging cell line (Phoenix Ampho) was transfected, using calcium phosphate precipitation, with the pQCXIN vector in which the gene encoding human CD38 was inserted. These cell lines are referred to as UM9-CD38 and L363-CD38. Cells were cultured in IMDM medium (Lonza, BE12-722F) (Wien-133) or RPMI 1640 medium (Lonza, BE12-115F) (all other cell lines), supplemented with 10% heat-inactivated cosmic calf serum (CCS) (Perbio, SH30087-03), 50 IU/ml penicillin and 50 μg/ml streptomycin (Lonza, DE17-603E). The culture medium for Daudi, Daudi-luc and Ramos cells was supplemented with 2mM L-glutamine (Lonza, BE17-605F) and 1 mM sodium pyruvate (Lonza, BE13-115E).

### Patient-derived MM cells

Mononuclear cells (MNC) from MM patients, isolated from the bone marrow, pleural fluid or blood, were obtained after informed consent and approval by the Medical Ethical Committee (University Medical Center, Utrecht, The Netherlands).

### Antibodies

Human IgG1 CD38 mAb DARA was generated by immunization in a HuMAb mouse and produced as recombinant protein as described previously.^12^ DARA F(ab’)_2_ fragments were prepared via pepsin digestion (Sigma-Aldrich, P7012). An IgG2 variant of DARA (DARA-IgG2) was constructed by cloning the variable region of the immunoglobulin heavy chain (V_H_) of DARA into a human IgG2 backbone. The human heavy chain construct was co-expressed with the appropriate original human kappa light chain. Fc mutants were generated by mutating the lysine residue at position 322 to alanine; these mutants are referred to as DARA-K322A and DARA-IgG2-K322A. The mutations were introduced as described previously.^16,19^ The human mAb IgG1 b12, specific for the HIV-1 gp120 envelope glycoprotein,^47^ was included in all experiments as an irrelevant mAb control. IgG concentrations were determined by A280 measurements.

### Bone marrow-derived mouse macrophage culture

Bone marrow was isolated by flushing the femurs of female SCID mice (C.B-17/Icr-Prkdc^scid^/Crl), purchased from Charles River, filtered and subsequently cultured for 7 d in DMEM (Lonza, BE12-079F) with 10% heat-inactivated CCS, 2 mM L-glutamine, 50 IU/ml penicillin, 50 μg/ml streptomycin (complete mφ medium) supplemented with 50 U/ml M-CSF (ProSpec, 315-02) as described previously.^16^ Mφ were detached with 0.5 mM EDTA (Versene, Gibco, 15040-033) and characterized by flow cytometry on a FACSCantoII (BD Biosciences) using F4/80-PE (Invitrogen, MF48004), CD80-PE (eBioscience, 12-0801), CD64-PE and CD32/16-FITC (BD Biosciences, 558455 and 553144, respectively) specific Ab.

### Monocyte-derived human macrophage culture

PBMC were isolated from buffy coats obtained from regular blood bank donations (after informed consent, Sanquin Blood Bank, Utrecht, The Netherlands) using density separation with Lymphocyte Separation Medium (Lonza, US17-829E), followed by washing with PBS (B. Braun, 3623140) Monocytes were isolated via negative selection from the PBMC fraction using the Dynabeads Untouched Human Monocytes isolation kit (Invitrogen, 113.50D). Isolated monocytes were cultured for 7 d in complete mφ medium supplemented with 10 ng/ml GM-CSF (Invitrogen, PHC2015). Mφ were detached with 0.1% trypsin-EDTA (Invitrogen, 15400-054) in PBS and characterized by flow cytometry (FACSCantoII) for staining with CD11b-PE (555388), CD32-FITC (552883), CD16-FITC (555406) from BD Biosciences and CD64-FITC (Biolegend, 305006).

### Flow cytometry

CD38 cell surface expression was quantified using mouse-anti-human CD38 antibodies (BD Biosciences, 555458) and the Qifi kit (DAKO, K0078), according to the manufacturer's guidelines. Samples were analyzed with flow cytometry (FACSCantoII).

### Antibody-dependent phagocytosis

Mφ were seeded either at 2.5 × 10^5^ cells per well into 24-well plates or 1 × 10^5^ cells per well into 96-well plates and allowed to adhere. Target cells were labeled with calcein-AM (Invitrogen, C-3100) and added to the mφ at an effector:target (E:T) ratio of ∼1:1 (mouse mφ) or ∼2:1 (human mφ) in the presence of 6.7 nM mAb (equals 1 μg/ml IgG1 or 0.7 μg/ml F(ab’)_2_ fragments). After 4 h incubation at 37°C/5% CO_2_ the supernatant containing non-phagocytosed target cells was collected. Mφ were detached with 0.1% trypsin-EDTA and subsequently added to the non-phagocytosed target cells. The pooled cells were kept at 4°C and stained with either anti-F4/80-PE (mouse mφ) or anti-CD11b-PE (human mφ) to identify mφ. Non-phagocytosed BL or MM target cells, either free or bound to macrophages, were detected using anti-CD19-APC (DAKO, C7224) or anti-CD138-APC (Beckman Coulter, PN A87787), respectively.

Antibody-dependent phagocytosis was assessed with flow cytometry (FACSCantoII), and quantified in 2 ways. First, as the percentage of mφ that had phagocytosed, determined using the percentage of F4/80^+^/calcein^+^ (mouse) or CD11b^+^/calcein^+^ (human) (double positive; DP) mφ. To exclude mφ only adhering to target cells (not engulfing), DP macrophages also positive for the BL or MM markers CD19 or CD138 were excluded from the DP population. For each sample, the total number of mφ in the sample (F4/80^+^ or CD11b^+^ cells) was used as 100% value. Second, phagocytosis was quantified by counting the number of remaining target cells (F4/80^-^ or CD11b^-^) with and without Ab treatment. To exclude elimination of target cells via additional killing mechanisms, like e.g. ADCC, which leads to loss of calcein through leakage of the plasma membrane, we included both calcein-negative and -positive cells when counting the number of remaining target cells. Elimination of target cells was calculated using the following formula:

% eliminated target cells=100−[remaining target cells after Ab treatmentremaining target cells without Ab treatment×100%]

The percentage DP mφ and the percentage of eliminated target cells showed substantial variation between experiments, most likely due to the use of different batches of cultured macrophages that originated from different donors. To allow comparisons between experiments with patient MM cells, Daudi cells were included in each experiment as an internal control. To correct for differences in levels of baseline phagocytosis between patient samples, we subtracted the percentages found with the irrelevant mAb control from the percentage observed in the presence of DARA. Results obtained using patient MM cells were normalized using the following formulas:

normalized DP mφ=100×patient sample(% DP with DARA−% DP with irrelevant mAb)Daudi(% DP with DARA−% DP with irrelevant mAb)

normalized eliminated target cells = 100×patient sample(% elimination with DARA−% elimination with irrelevant mAb)Daudi(% elimination with DARA−% elimination with irrelevant mAb)

### Live cell imaging

For live cell imaging, target cells and mouse mφ were incubated (1–10 × 10^6^ cells/ml) in HBSS (Gibco, 24020-091) or complete mφ medium, supplemented with 25 μg/ml of the fluorescent dyes DiB (Biotium, 60036) or 3,3′-dioctadecyloxacarbocyanine perchlorate (DiO, Molecular Probes Inc. D-275), respectively, for 30 minutes at 37°C, and subsequently washed 3 times with complete mφ medium. Mφ were seeded at 2 × 10^5^ cells/well into 8 wells ibiTreat μ-Slides (IBIDI, 80826) and allowed to adhere O/N. Real-time phagocytosis assays were performed with indicated E:T ratios at a fixed mAb concentration and imaged with an Olympus CellR real-time live-imaging station (type IX81, UPLFLN 40 × O/1.3 lens). Pictures were taken every 20 seconds with an Olympus ColorView II camera for 30 minutes.

### Mouse tumor xenograft models

Experiments were performed with 8–12 weeks old female SCID-BEIGE mice (C.B-17/IcrHsd-Prkdc^scid^Lys^bg^), purchased from Harlan. Mice were housed in a barrier unit of the Central Laboratory Animal Facility (Utrecht, The Netherlands) and kept in individually ventilated cages with water and food provided *ad libitum*. Mice were checked at least twice a week for clinical signs of disease and discomfort. All experiments were approved by the Utrecht University animal ethics committee. Subcutaneous tumors were induced by inoculation of 20 × 10^6^ Daudi-luc cells in BD Matrigel Basement Membrane Matrix High Concentration (BD Biosciences, 354248) in the right flank of mice and tumor volumes were calculated from digital caliper measurements as 0.52 × length × width^2^ (in mm^3^). Experimental leukemia was induced by injecting 2.5 × 10^6^ Daudi-luc cells into the tail vein. At weekly intervals, tumor growth was assessed using bioluminescence imaging on a Photon Imager (Biospace Lab). Before imaging, mice were anaesthetized via isoflurane and synthetic d-luciferin (Biothema, BT11–1000) was given i.p. at a dose of 125 mg/kg, M3 vision software (Biospace Lab) was used for image analysis. MAb were injected i.p. at day 0, after tumor cell inoculation, at indicated dosing levels. During the study, heparinized blood samples were taken for determination of IgG levels in plasma using a Behring Nephelometer II (Siemens Healthcare Diagnostics).

### Statistical analysis

Data analysis was performed using GraphPad Prism 5.0 (Graphpad) and PASW Statistics 18.0 (SPSS Inc.) software. Data were reported as mean ± SEM. Differences between groups were analyzed by one-way ANOVA with Bonferroni's multiple comparison post-test (Prism) or Mantle-Cox log-rank test (PASW).
