# Supplementary material for: Antibody-mediated phagocytosis contributes to the anti-tumor activity of the therapeutic antibody daratumumab in lymphoma and multiple myeloma
Source: MAbs. 2015 Mar 11;7(2):311–20. doi: 10.1080/19420862.2015.1007813 (PMC4622648; doi:10.1080/19420862.2015.1007813)
Supplement: suppl_materail_KMAB_1007813.zip [file kmab-07-02-1007813-s001.zip › suppl fig 1.pdf]

Supplemental Figure 1

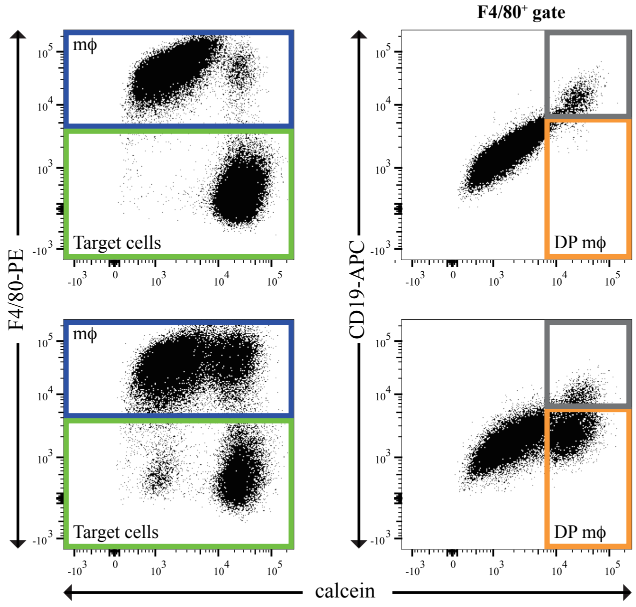

Supplemental figure 1. Representative flow cytometry plot of Daudi cells co-cultured with mouse mφ in the presence of irrelevant mAb control (upper panels) or DARA (lower panels). F4/80 is used as marker to identify the mφ, which are in the blue gate (F4/80+). Target cells were identified by lack of F4/80- target expression (green gate). The right panels show further differentiation within the F4/80+ gate, between mφ that phagocytosed (F480+,calcein+,CD19-; orange gate, "double positive mφ") and those which have not phagocytosed target cells (F4/80+, calcein-, CD19-). The F4/80+,calcein+,CD19+ population (gray gate) represents mφ to which calcein+CD19+ target cells have adhered. Because it is unclear whether these macrophages have also ingested target cells, they were considered phagocytosis-negative and excluded from the population of double positive macrophages.
