# Supplementary material for: Antibody-mediated phagocytosis contributes to the anti-tumor activity of the therapeutic antibody daratumumab in lymphoma and multiple myeloma
Source: MAbs. 2015 Mar 11;7(2):311–20. doi: 10.1080/19420862.2015.1007813 (PMC4622648; doi:10.1080/19420862.2015.1007813)
Supplement: suppl_materail_KMAB_1007813.zip [file kmab-07-02-1007813-s001.zip › suppl fig 2.pdf]

Supplemental Figure 2

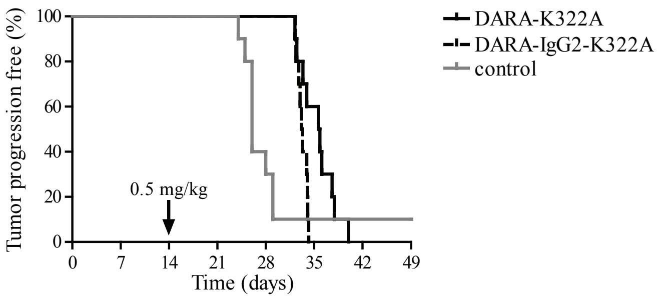

Supplemental figure 2. DARA is more potent than DARA-IgG2-K322A in a therapeutic i.v. Daudi-luc SCID-BEIGE xenograft model. Kaplan-Meier plot showing time to tumor progression (cutoff set at bioluminescence > 500 000 cpm) for mice that had been inoculated i.v. with 2.5×10^6 Daudi-luc cells (10 mice per group). Mice were treated with 10 µg mAb per mouse (~0.5 mg/kg) at day 14. Tumor progression was significantly slower in DARA-K322A-treated vs DARA-IgG2-K322A-treated mice ($p<0.012$ Mantle-Cox log-rank test at time to progression).
