# Supplementary material for: Antibody-mediated phagocytosis contributes to the anti-tumor activity of the therapeutic antibody daratumumab in lymphoma and multiple myeloma
Source: MAbs. 2015 Mar 11;7(2):311–20. doi: 10.1080/19420862.2015.1007813 (PMC4622648; doi:10.1080/19420862.2015.1007813)
Supplement: suppl_materail_KMAB_1007813.zip [file kmab-07-02-1007813-s001.zip › suppl fig 3.pdf]

Supplemental Figure 3

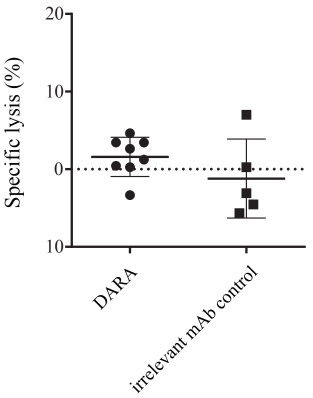

Supplemental figure 3. Absence of mφ-mediated ADCC induction by DARA on Daudi cells. Chromium-release assay in which Daudi cells, labeled with 100 µCi $Na_2{}^{51}CrO_4$ (Perkin-Elmer, NEZ030002MC), were pre-incubated with 10 µg/ml DARA or irrelevant mAb control for 15 min at room temperature. Medium was included as a negative control, Triton-X-100 as a positive control for maximal release. Subsequently, bone marrow derived mouse mφ were added at an effector to target ratio of 20:1 and incubated for 24h at 37 ºC. Supernatants were collected and chromium release was measured in gamma counter. Percentage specific lysis was calculated using the following formula: (release after Ab treatment [cpm] – release negative control [cpm]) / (release positive control [cpm] - release negative control [cpm]). Each bar shows mean ± S.D., a representative experiment is shown; similar results were obtained with macrophages from wild-type and CD16 knock-out mice).
